# Lumbar and Thoracic Vertebrae Segmentation in CT Scans Using a 3D Multi-Object Localization and Segmentation CNN

**DOI:** 10.3390/tomography10050057

**Published:** 2024-05-13

**Authors:** Xiaofan Xiong, Stephen A. Graves, Brandie A. Gross, John M. Buatti, Reinhard R. Beichel

**Affiliations:** 1 Department of Biomedical Engineering, The University of Iowa, Iowa City, IA 52242, USA; xiaofan-xiong@uiowa.edu; 2 Department of Radiology, The University of Iowa, Iowa City, IA 52242, USA; stephen-a-graves@uiowa.edu; 3 Department of Radiation Oncology, University of Iowa Hospitals and Clinics, Iowa City, IA 52242, USA; brandie-gross@uiowa.edu (B.A.G.); john-buatti@uiowa.edu (J.M.B.); 4 Department of Electrical and Computer Engineering, The University of Iowa, Iowa City, IA 52242, USA

**Keywords:** vertebrae, localization, segmentation, CT

## Abstract

Radiation treatment of cancers like prostate or cervix cancer requires considering nearby bone structures like vertebrae. In this work, we present and validate a novel automated method for the 3D segmentation of individual lumbar and thoracic vertebra in computed tomography (CT) scans. It is based on a single, low-complexity convolutional neural network (CNN) architecture which works well even if little application-specific training data are available. It is based on volume patch-based processing, enabling the handling of arbitrary scan sizes. For each patch, it performs segmentation and an estimation of up to three vertebrae center locations in one step, which enables utilizing an advanced post-processing scheme to achieve high segmentation accuracy, as required for clinical use. Overall, 1763 vertebrae were used for the performance assessment. On 26 CT scans acquired for standard radiation treatment planning, a Dice coefficient of 0.921 ± 0.047 (mean ± standard deviation) and a signed distance error of 0.271 ± 0.748 mm was achieved. On the large-sized publicly available VerSe2020 data set with 129 CT scans depicting lumbar and thoracic vertebrae, the overall Dice coefficient was 0.940 ± 0.065 and the signed distance error was 0.109 ± 0.301 mm. A comparison to other methods that have been validated on VerSe data showed that our approach achieved a better overall segmentation performance.

## 1. Introduction

The segmentation of vertebrae in CT scans is important for various clinical applications, including the identification and assessment of spine abnormalities, modeling of biomechanics, or planning of spine interventions. In the context of bone marrow sparing using intensity-modulated radiation therapy (BMS IMRT) and dose calculation from radiopharmaceutical therapy, the accurate segmentation of lumbar vertebrae and pelvic bone structures in computed tomography (CT) scans can be used as a surrogate to define bone marrow at risk for the purposes of radiation therapy planning and has been illustrated using [F-18] fluorothymidine (FLT) positron emission tomography and computed tomography (PET-CT) images [1]. However, manually delineating vertebrae is a time-consuming (hours-long) task that is prone to inconsistencies. Therefore, developing automatic segmentation methods will help to improve the efficiency and consistency of the segmentation process needed for radiation dose calculations for the bone marrow.

### 1.1. Related Work

For the automated segmentation of vertebrae, several approaches have been proposed, but most approaches consist of two main stages: the identification (sometimes split into localization and labeling) of vertebrae and segmentation of individual vertebra. The identification step can be performed by manually placing seeds within the vertebral body [2] or drawing a bounding box to restrict the search volume [3]. Additional automated techniques such as first extracting the spine curvature followed by detecting vertebrae by locating the inter-vertebral disks [4,5] and utilizing unique characteristics of vertebrae like shape [6] and symmetry [7] have also been proposed. Machine learning techniques such as random forest [8,9] were also utilized and achieved promising results. Recently, more convolutional neural network (CNN)-based methods were proposed. Sekuboyina et al. [10] proposed to label maximum-intensity projections of the spine in sagittal and coronal planes. Their approach utilizes an adversarial network training regime to encode an anatomical prior. Mader et al. [11] used a 3D CNN to regress the heatmap responses of vertebrae locations followed by fine-tuning using regression trees. McCoaut et al. [12] proposed an approach for vertebrae detection and localization based on two-stage CNNs and dense annotations. For segmentation, traditional methods such as region growing [13] and graph cut [14] were utilized but suffered from leakage and holes because of ambiguous edges and the poor regional homogeneity of vertebrae. Model-based methods involve fitting a shape prior to a target region by deforming it based on local image features [15,16,17]. With the emergence of deep learning in image analysis, more recent methods proposed deep CNNs for vertebrae segmentation.

An overview of state-of-the-art vertebrae identification and segmentation methods can be found in the VerSe’20 and VerSe’19 challenges summary paper by Sekuboyina et al. [18], which provides a description and comparison of 24 approaches. Furthermore, these challenges resulted in a large and publicly available data collection for vertebrae segmentation performance analysis and comparison. Other existing data collections that are frequently utilized for this purpose include CSI-Seg 2014 [19,20] and xVertSeg [16], but they are of limited relevance due to their small test data set size of ten and ten cases, respectively. Furthermore, these data sets do not fully cover the age range of the (older) oncology-relevant populations. Consequently, these data sets are not representative of the broad clinical data spectrum and can result in performance estimation biases. Thus, VerSe data sets currently represent the most relevant CT scan collection for estimating real-world clinical performance. Out of the 24 methods described in the summary paper [18], 21 utilize some form of a deep-learning-based approach. Furthermore, the majority of approaches deploy at least two processing stages—one for localizing vertebrae and one for actual vertebrae segmentation, and typically some form of CNN-based approach is utilized to implement these processing stages. For more details regarding the pros and cons of the 24 methods as well as the implementation details, the reader is referred to [18]. The methods developed by Payer et al. [21], Chen D. et al. [18], and Lessmann et al. [22] stand out as high-performing, based mostly on the segmentation performance analysis presented in [18], and are discussed in more detail below.

Payer et al. [21] proposed a multi-stage approach. First, a 3D U-Net is utilized to generate a heatmap of the spinal centerline. Second, individual vertebrae are localized and identified by using a SpatialConfig-Net. Third, all vertebrae are segmented individually. Note that in a second version of their approach, they added a Markov Random field to post-process the output of the vertebrae localization stage. Chen D. et al. also utilize a multi-stage approach [18]. First, a 3D U-Net is utilized to coarsely localize the spine. Second, a U-Net performs a patch-wise binary segmentation of vertebrae. Third, a 3D ResNet model is used to label vertebrae based on the segmentation result as well as CT image data, and a Deep Reasoning Network [23] is utilized to impose the anatomical correctness of the sequence of vertebrae. Lessmann et al. [22] proposed a single-stage approach for segmentation in which volume patches are being processed to generate vertebrae segmentation. For this purpose, a CNN is used to iteratively identify and segment vertebrae in patches. This process is started from the bottom of the CT scan and iteratively follows the spine structure. Subsequently, an additional network is utilized to detect the first cervical and thoracic vertebrae [18].

Some recently published methods that were not part of a VerSe challenge are discussed in the following paragraph. Cheng et al. [24] propose a two-stage approach. First, a Dense-U-Net is utilized to process slabs of two cross-sectional CT images to localize vertebrae. Second, a 3D-Dense-U-Net is used to segment individual vertebrae. The authors claim that their method is fully automated. However, because the Dense-U-Net in the first stage expects cross-sectional images of 128×128×9 voxels, it is unclear how their method can automatically process volumes of different sizes. For lumbar vertebrae segmentation, Lu et al. [25] utilize a two-stage approach. First, a U-Net-based approach is utilized to locate the region of the lumbar spine. Second, a 3D XUNet is applied to segment lumbar vertebrae. Note that the first processing stage expects a fixed-sized input volume. Thus, this is an issue when processing arbitrarily sized scans. Wu et al. [26] also report a method for lumbar vertebrae segmentation. Their approach is based on utilizing the fusion envelope of 2D hybrid visual projection images to locate lumbar vertebrae. Once located, a 3D U-Net is utilized for segmentation of the vertebrae. Because their approach was not assessed on a large, diverse, and publicly available data set like VerSe, it is unclear how their approach would compare to other methods on relevant image data. Qadri et al. [27] propose an approach where axial CT slices are processed by dividing them into overlapping image patches, which are then fed into a classifier to determine whether a given patch represents a vertebra or not. For this purpose, a stacked sparse autoencoder in combination with a random under-sampling module is utilized. The use of 32×32 image (2D) patches might be a limiting factor, as the full 3D context provided by volumetric scans is not utilized. Furthermore, vertebrae are not segmented and labeled individually. Thus, individual vertebrae might be fused. The work published by Meng et al. [28] is focused on improving the performance of transitional vertebrae identification. For this purpose, they propose an iterative approach in which in each cycle, individual vertebrae are localized, segmented, and identified using deep networks in combination with statistical priors to enforce consistency. A potential advantage of their approach is that if the anatomic consistency criteria were not met in a local region, it is reported, which is helpful for manual quality control. Recently, vision transformers (ViTs) have shown good performance on computer vision applications [29], and You et al. [30] propose a single-staged ViT-based model for vertebrae segmentation in CT scans. It utilizes a U-Net-like structure with an embedded ViT component, which is enhanced by edge detection and a global information extraction block. While proposing an interesting network architecture, its performance on VerSe’20 and VerSe’19 challenge data remains below the top-performing multiple-stage approaches [18]. Because of the use of a ViT, it seems likely that a substantially larger training data set will be needed to show a comparable performance.

In summary, most better-performing methods use a multiple-stage approach that is implemented by multiple network structures. CT volumes of varied sizes can be an issue for some approaches because the utilized CNNs expect an input of a fixed size (e.g., Cheng et al. [24]), and some manual intervention (cropping or resampling) will be needed. Most methods deploy a 3D CNN for segmenting individual vertebrae after localization, which provides one subvolume per vertebrae. While this approach works fine for most cases, low contrast and/or vertebrae in close proximity due to degeneration, typical of older cancer patients, can cause segmentation errors that need manual correction. However, more elaborate strategies to correct segmentation errors of the network are typically not explored.

### 1.2. Contribution

The motivation behind the presented work is to facilitate two applications: (a) BMS IMRT in the context of treating pelvic tumors like prostate and cervix tumors and (b) marrow-specific radiation dosimetry, enabling advanced treatment methods in radiopharmaceutical therapy. Consequently, bone structures like the pelvis as well as lumbar and thoracic vertebrae are of special interest. In addition, focus has been put on the ease of integration into clinical workflow. Therefore, the combination of several key characteristics distinguishes our approach from existing methods and makes it especially well-suited for our application domain. Specifically, our main contributions include:

(i) the use of a single, low-complexity network with no “duplicate” network components, thereby avoiding a multiple CNN approach where location and segmentation networks are required to learn object appearance separately;
(ii) the ability to process arbitrary-sized CT volumes by processing small, overlapping volume patches;
(iii) center location awareness of vertebrae within the processed volume patch, enabling advanced post-processing strategies needed to achieve high accuracy and, thus, clinical utility;
(iv) the demonstration of the separate segmentation of vertebrae boundary and body (i.e., to facilitate the accurate calculation of bone marrow dose in radiopharmaceutical therapy);
(v) achieving excellent performance (a Dice coefficient larger than 0.9) with even small training data sets, which is important for research applications with specialized imaging protocols and a limited number of scans available.

Note that, while achieving characteristics (ii) and (iii) at the same time seems to be counter-intuitive due to the limited field of view, the presented work successfully demonstrates the feasibility of this task. Furthermore, this work is a first step toward developing a volume patch-based center-location-aware multi-organ segmentation method (i.e., multiple different objects). Thus, our approach has promise beyond the studied application domain.

We assess the performance of our approach on two data sets. The first is a small-sized data set that is directly representative of image data used in the context of BMS IMRT (Section 3.1.1), and it consists of consistently obtained radiation treatment simulation CT images. In contrast, the second data set is based on a large-sized, diverse, and multi-institutional image data set that was utilized as a part of the VerSe’20 challenge and enables comparing our approach with other vertebrae segmentation methods (Section 3.1.2). By using a multi-institutional data set, it validates the robustness of the approach across a range of image acquisition parameters and imaging devices.

## 2. Methods

**Figure 1:**
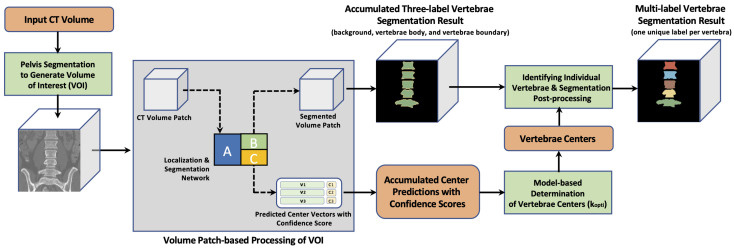
Method overview.

Figure 1 provides an overview of our method. Our novel approach uses a single CNN. This is in contrast to the currently top-performing approaches, which are based on utilizing multiple networks, as discussed in Section 1.1. The advantages of using a single network include the avoidance of redundancy leading to reduced network complexity, reduced training times, and achieving a satisfactory performance even with smaller training data sets. We note that these characteristics are achieved without a compromise in the segmentation performance when compared to multi-network approaches. Our network has two outputs and processes volume patches of the input CT scan. It is used to (a) segment all partially or completely visible vertebrae present in the patch and (b) estimate up to three associated vertebrae center locations (Figure 1). Both results are accumulated and analyzed to (a) find the number and location of individual vertebrae as well as (b) post-process segmentations to produce more robust segmentations of individual vertebrae. Our patch-based approach addresses the issue of processing vastly different image volumes.

The utilized CNN structure builds on our previous work on the combined localization and segmentation of the pelvis [31] (i.e., a single structure). In this context, a key challenge is that within a volume patch, none, one, two, or several vertebrae can be visible, compared to the single structure case described in [31]. Voting for the center location of just one of the visible vertebra structures (i.e., the largest vertebrae part visible in the volume path) has the disadvantage of reduced robustness because individual vertebrae might be only covered by a small number of volume patches. In this work, we address this issue by predicting three vertebrae locations per volume patch. In addition, we introduce a confidence value for each of the predictions, enabling the correct modeling of situations where only one or two vertebrae are visible. Once all volume patches of interest are processed, the predicted centers with associated confidence values are accumulated, resulting in a more robust localization of vertebrae, which is important for enabling segmentation post-processing. Thus, in this work, we successfully demonstrate how to extend the combined localization and segmentation approach from one unique object (pelvis) to multiple similar objects (vertebrae) and describe advanced post-processing steps that are enabled by the gained information about neighboring vertebrae.

In the following Section 2.1, Section 2.2 and Section 2.3, the utilized network as well as its training and application are described. The model-based determination of vertebrae centers is discussed in Section 2.4. Subsequently, the approach for identifying individual vertebrae and segmentation post-processing is presented in Section 2.5.

### 2.1. Combined Localization and Segmentation Network for Multiple Objects

Our approach to vertebrae segmentation assumes that the pelvis was already located by utilizing our method presented in [31]. This enables us to specify a rough volume of interest (VOI) for lumbar and thoracic vertebrae. Subsequently, the task is to individually localize and segment all vertebrae inside the VOI. For this purpose, we use volume patch-based processing with a CNN, consisting of a localization and segmentation component. The rationale behind this approach is as follows. If trained correctly, the segmentation CNN is already quite selective. Thus, if several voxels of a processed volume patch are labeled as the target structure (vertebrae), then we can utilize the patch to predict their location. Note that a volume patch can contain several instances or parts of different vertebrae. Therefore, multiple location predictions are typically needed. Once all volume patches have been processed, a refined prediction about the center location of individual vertebrae can be made, which is helpful for separating neighboring vertebrae that can be hard to differentiate.

**Figure 2:**
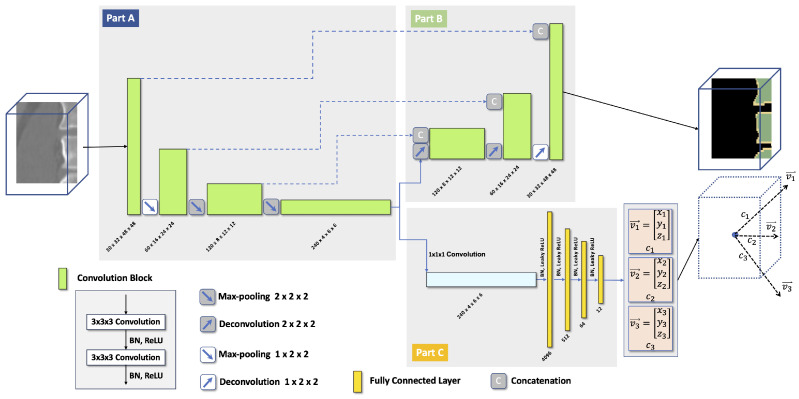
The network structure consists of (a) a segmentation component (parts A and B) and (b) a regression component for localization (parts A and C).

The utilized network consists of two main components: (a) a CNN-based component for segmentation (parts A and B in Figure 2) and (b) a regression component for localization (parts A and C in Figure 2). Note that part A is shared by both network components because learning object appearance is important for segmentation and localization. Therefore, our network structure is more efficient compared to approaches employing two or more separate networks. Both network components are further discussed below.

**(a) Segmentation component** We utilize a 3D U-Net [32] for the intrinsic vertebrae segmentation with two modifications to improve performance: the ReLU activation has been replaced by Leaky ReLU and batch normalization by an instance normalization [33]. Note that for our approach, other network variants with a contracting path followed by an expanding path are also suitable candidates. Thus, if a better-performing network variant becomes available, it can be used to replace the utilized CNN.

**Figure 3:**
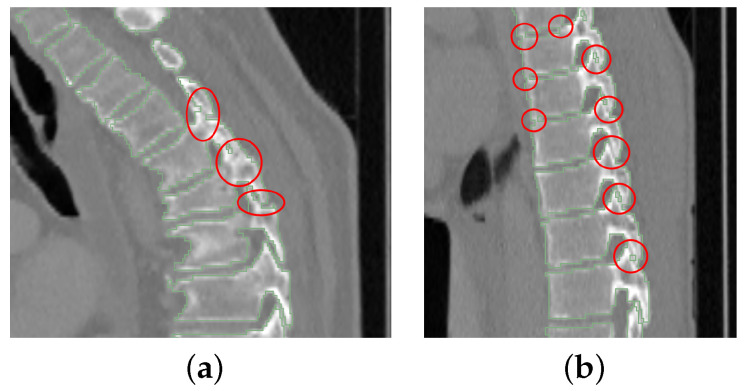
Two examples of segmented upper thoracic vertebrae when some vertebrae are connected (red circles). (**a**) Some processes are connected. (**b**) Some vertebral bodies and processes have connections.

**Figure 4:**
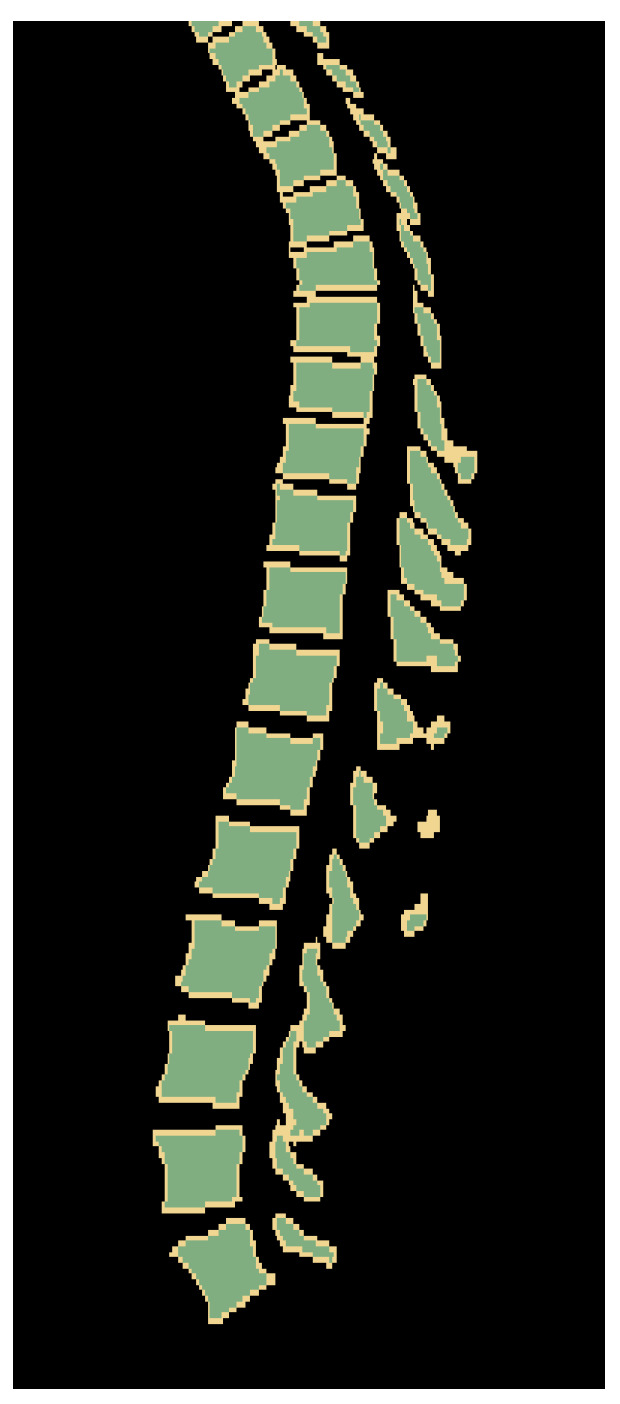
The three-label segmentation containing the inner body of vertebra (green), the vertebra boundary (yellow), and the background (black).

The used network has four tiers. Furthermore, one problem with vertebrae segmentation is leakage between individual vertebra segmentations, resulting in label maps with partially connected neighboring vertebrae, especially nearby spinal processes. This happens more frequently for upper thoracic vertebrae as they tend to be significantly smaller and located closer to each other (Figure 3). To solve this problem, we use three labels to represent a segmentation result: the inner body of vertebra, vertebra boundary, and background. An example is shown in Figure 4.

**(b) Localization component** For the localization component (Figure 2), we use a convolutional regression network that takes the CNN bottleneck data as input. It contains one 1×1×1 convolutional layer followed by four fully connected layers. A Leaky ReLU is used as the activation function, and batch normalization is applied in each layer. The network output is a 1×12 vector because the localization component is designed to localize three objects. In addition, a localization confidence score is introduced for each object. To be specific, given an input volume patch, it can contain from one to more than three vertebrae. To be able to use the same localization output vector size, we choose to always predict the locations of three consecutive vertebrae using three 1×3 vectors, pointing from the volume patch center to the center of mass of each vertebral body, which is generated by applying morphological opening operations on a ground truth vertebra mask to remove the processes. The three centers are referred to as top-center, mid-center, and bottom-center, and each center is associated with a confidence score. Combined, the three 1×3 vectors and the three confidence scores form the 1×12 output vector. Details about how the three target vertebrae are chosen based on the number of visible vertebrae inside the volume patch are explained in Section 2.2.

### 2.2. Network Training

For the two data sets, there is a difference between the cross-validation setup used for training and performance evaluation. For the Iowa data, the 26 planning CT scans with ground truth label maps are split into three folds with roughly the same number of scans so that three independent experiments can be implemented. In each experiment, two folds are used for training and the other fold as the independent testing data. For the two training folds, a 5-fold cross-validation approach is used, where approximately 4/5 of the data are used for training and the left out 1/5 as validation set. Therefore, five networks can be trained in each experiment. In the case of the VerSe2020 image data, a dedicated training data set is provided (Section 3.1.2). Thus, a 5-fold cross-validation approach is utilized for the training data, then the five trained models are applied on two independent test sets (Section 3.1.2) for the performance assessment.

Network training is volume-patch-based, and the soft Dice coefficient loss is used as a loss function. Furthermore, network weights are initialized using the Kaiming initialization with normal distribution. To increase the training set size, several patch-based augmentations are applied, including random rotation (range: ±15 degrees per axis) and random scaling (range: ±25%). One important extra step is that all multi-label ground truth segmentations (i.e., one label per vertebra) are transformed into three labels per vertebra to provide body, boundary, and background information for segmentation CNN training. The extracted CT volume patches are of 32×48×48 voxels with a 50% overlap in all three directions. For training of the segmentation component, a batch size of 32 was used. This selection is optimized for the available memory of the graphics processing unit (GPU; GeForce GTX 1080 Ti with 11 GB memory, NVIDIA Corp., Santa Clara, CA, USA). A smaller batch size showed a negligible difference in performance but will result in a longer training time. In each batch, at least half of the volume patches are chosen to include a part of the lumbar vertebrae while the other half is randomly selected. The motivation is to create a balanced representation of patches with and without vertebrae voxels, which leads to improved convergence in network training.

**Figure 5:**
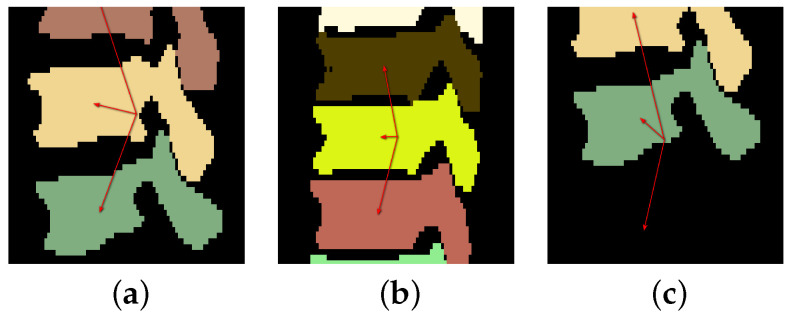
Examples of volume patches with corresponding localization vectors (red arrows). (**a**) Three vertebrae are visible in the patch, and each is indicated by a vector. (**b**) Five vertebrae are visible in the patch and only the middle three are indicated by vectors. (**c**) Only two vertebrae are visible in the patch, but an additional calculated (low confidence) center at the bottom is also indicated by a vector.

As mentioned in part (b) of Section 2.1, the output of the localization component is a 1×12 vector. Thus, a 1×12 ground truth vector is required for each volume patch, including three 1×3 vectors pointing from the patch center to three consecutive vertebra centers and three corresponding confidence scores. The motivation for this design choice is as follows. While the output vector of the localization network must be of a fixed size, the number of vertebrae (partially) visible can vary from one to five for the selected size of the volume patch. However, in most cases, three vertebrae will be visible. This number can be less at the transition from L5 to the pelvis or at the superior start of the CT scan. To account for such situations, a plausible vertebrae location is assumed based on the visible middle and adjacent vertebrae. In addition, the corresponding confidence value will be set to zero. In the case of four or five visible vertebrae parts, the focus is on the vertebrae located most prominently in the center of the volume patch. Some examples of the volume patches with corresponding location vectors are shown in Figure 5. The approach for selecting the center vertebrae based on the visibility of vertebrae is as follows.

(i) One vertebra in patch: The center of the single vertebra is regarded as the mid-center.
(ii) Two vertebrae in patch: The center of the lower vertebra is regarded as the mid-center.
(iii) Three vertebrae in patch: The center of the middle vertebra is regarded as the mid-center.
(iv) More than three vertebrae in patch: First, the confidence score of all vertebrae partially inside the volume patch is sorted, and the one with the lowest score is excluded. This process is repeated until only three with the largest confidence scores are left. Then, the center of the middle vertebra of the three remaining is regarded as the mid-center.

An example where the top arrow is pointing to a calculated center instead of a real one is given in Figure 5a.

The confidence score is defined by the percentage of each vertebra inside the volume patch. For example, if a volume patch contains L1 to L3 with each having 15%, 25%, and 10%, respectively, of their total volume inside the volume patch, the three vectors will receive a confidence score of 0.15, 0.25, and 0.1, respectively. When the vertebra being pointed to is not in the volume patch (e.g., the number of vertebrae in a patch ≤ 2), the corresponding confidence score is set to 0.

For training the localization component, each of the five trained segmentation components in each experiment is first applied to CT scans of the corresponding training and validation folds to generate bottleneck data. However, only when a segmented volume patch has ≥15% of the typical (average) vertebra volume will its corresponding bottleneck data be used for the training and validation of the localization component. The selected level of 15% is a trade-off between the relevance of the volume patch for the localization and number of volume patches available for network training. A constant learning rate of 1 × 10^−4^ is used, and the epoch number is set to 400, which is sufficient for proper network convergence. A smaller number of iterations may not result in convergence, while a larger number will increase training time without a meaningful performance gain. Network weights are initialized using the Kaiming uniform distribution, and the loss function is a mean square error loss.

### 2.3. Network Application

**Figure 6:**
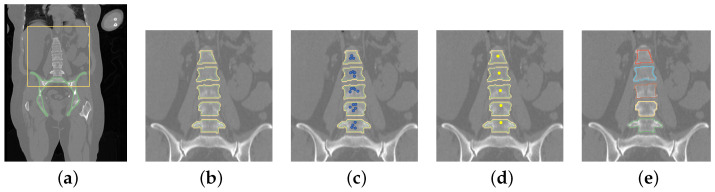
Workflow of the combined localization and segmentation network for a scan from the Iowa data set (see Section 2.3 and Section 2.5 for details).

The approach to utilizing the network is described in detail below and illustrated in Figure 6.

#### 2.3.1. VOI Generation

Our pelvis segmentation approach [31] is applied to all CT scans. For the Iowa data set (Section 3.1.1), all details regarding network training and application are as described in [31] (approach “single”). In the case of the VerSe2020 data (Section 3.1.2), the trained model as described in [31] (the “single” approach model with the lowest error score) was applied, because no pelvis reference segmentation is available for VerSe2020 CT scans.

After the pelvis is segmented, the VOI of vertebrae is straightforward to define (Figure 6a). Specifically, the center of mass of the segmented pelvis is used as the bottom center, based on which a VOI is cropped for vertebrae segmentation (the yellow rectangle in Figure 6a). The VOI size is different for the two data sets as the segmentation targets are different. Specifically, the crop size is 256×256×128 voxels for the Iowa data set. For the VerSe2020 data, the crop size is 320×320 voxels in the axial plane and the vertical size is dependent on the location of T1 if T1 is included in the scan; otherwise, it is dependent on the top voxel of the scan. Note that the axial crop size for the VerSe2020 data set is larger than the Iowa data set to cover the extra spine curvature when thoracic vertebrae are included. Both sizes are chosen so that all vertebrae to be segmented are covered. If the cropped VOI is outside the scan, the portions outside the scan are ignored.

**Figure 7:**
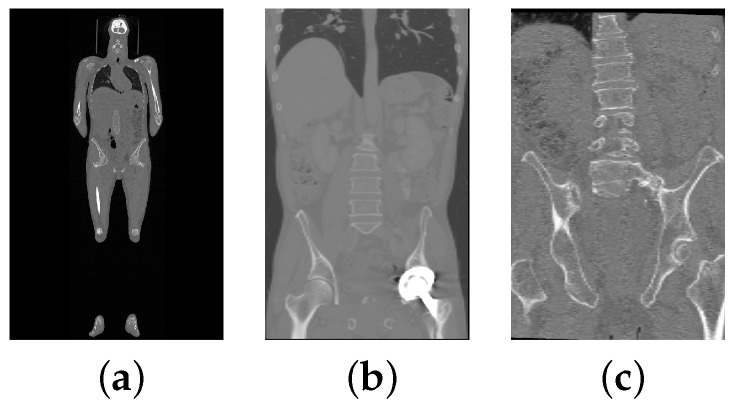
Examples of failed cases. (**a**) A scan with 512×512×1923 voxel that is too large to fit into GPU RAM. (**b**) Pelvis with an implant. (**c**) A scan with no obvious reason for failure.

In some cases, the CT scans in VerSe2020 do not or only partially show the pelvis, because this was not relevant for the vertebrae segmentation challenge. Also, for some VerSe2020 CT scans that fully depict the pelvis, the segmentation failed, because the CT scan is too large for the available GPU memory after resampling (Figure 7a) or because the appearance is altered by an implant (Figure 7b). However, there are also cases where the reason for failure is not obvious (Figure 7c). A potential explanation is that the pelvis model was trained on CT scans that are different from VerSe2020 images. Table 1 summarizes the frequency of cases with issues. For those cases, the following steps were performed, because the focus is on assessing the vertebrae segmentation performance. If there was no pelvis visible in a scan, cropping started from the bottom center of the scan instead of the pelvis center of mass. For cases with a partially imaged pelvis or pelvis segmentation failure (e.g., due to implants), the same procedure was applied, but additionally, an axial plane was identified manually to specify the lower boundary of the VOI. Furthermore, for the performance analysis, the results will be reported in two categories: (A) cases where the pelvis segmentation succeeded and (B) cases where the pelvis segmentation failed combined with cases where the pelvis is only partially or not imaged.

#### 2.3.2. Vertebrae Segmentation

Vertebrae are segmented inside the VOI by utilizing the segmentation component of the network in a patch-based fashion with a 50 % overlap in each direction. Subsequently, all segmentation results are combined to form the vertebrae segmentation. An example is shown in Figure 6b, where the yellow label represents the boundary and the green label represents the inner body.

#### 2.3.3. Vertebra Center Prediction

Each volume patch inside the VOI with more than 15% of vertebra voxels is further analyzed by feeding the corresponding segmentation CNN bottleneck data into the localization component of the network (Figure 2). Subsequently, each patch that meets this criterion is used to generate the location vector for three vertebrae combined with corresponding confidence scores (predicted vertebra centers are shown as blue points in Figure 6c).

### 2.4. Model-Based Determination of Vertebrae Centers

Using the accumulated center predictions as points and confidence scores as corresponding weights, a weighted k-means clustering algorithm is then applied to calculate final vertebra centers. Note that all the predicted centers inside the segmented pelvis are considered outliers and excluded before the k-means clustering. Because there can be different numbers of vertebrae in the VOI, a value $k_{opti}$ has to be found for the k-means algorithm to produce the correct number of center predictions. Thus, an objective function has to be defined to select $k_{opti}$, given a range of *k* values. For this purpose, the available VerSe2020 training data set was utilized because it includes lumbar and thoracic vertebrae.

**Figure 8:**
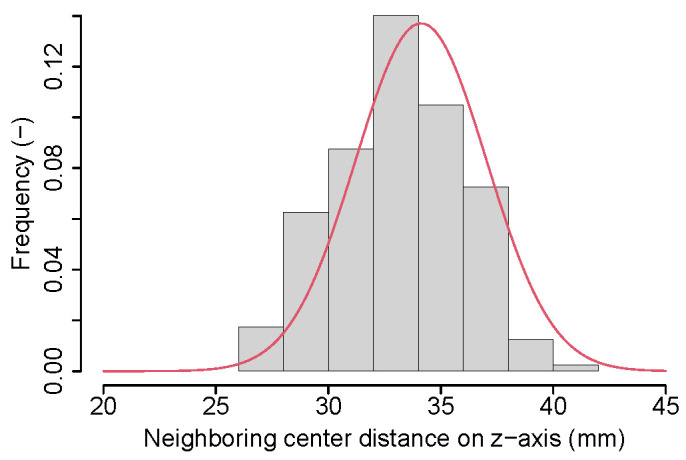
A Gaussian distribution (red curve) fitted to the histogram of the distance between each pair of neighboring vertebral centers projected on the z-axis across the training data set. Only lumbar vertebrae were considered.

**Figure 9:**
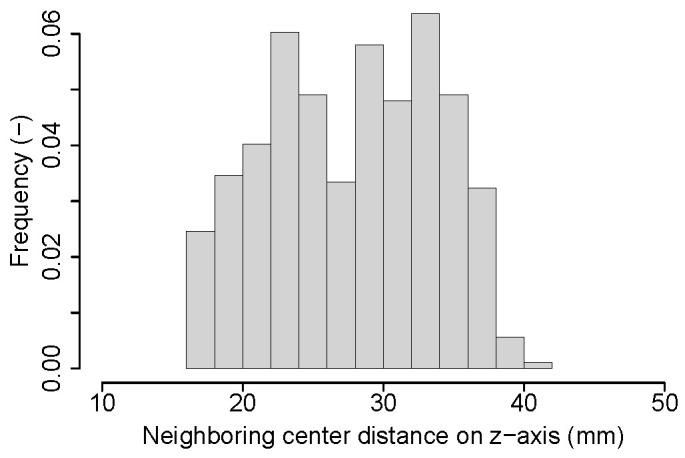
The histogram of the distance between each pair of neighboring vertebral centers projected on the z-axis across the training data set. Both lumbar and thoracic vertebrae were included.

**Figure 10:**
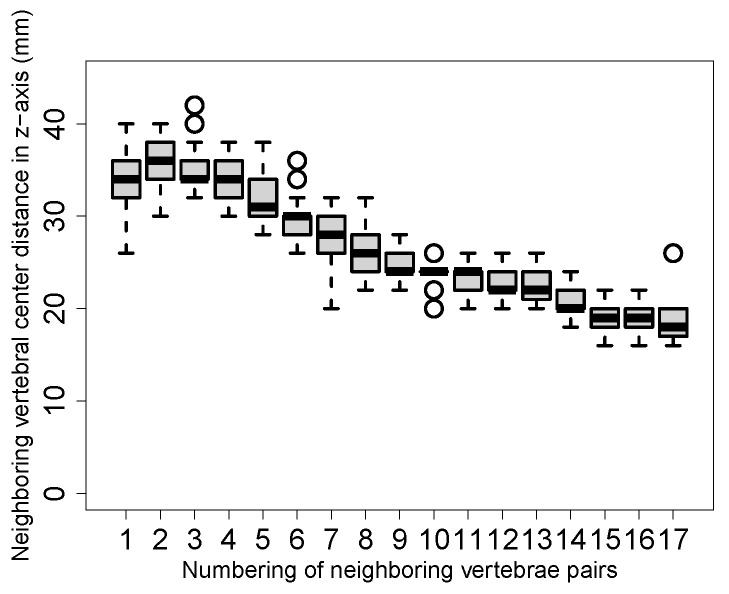
Boxplot of the distance between each individual pair of neighboring vertebral centers projected on the z-axis. Both lumbar and thoracic vertebrae are included. The number 1 on the x-axis represents the distance between the lowest two vertebrae (e.g., L5 and L4), the number increases for upper vertebrae, and the number 17 indicates the distance between the upper-most two vertebrae (e.g., T1 and T2).

**Figure 11:**
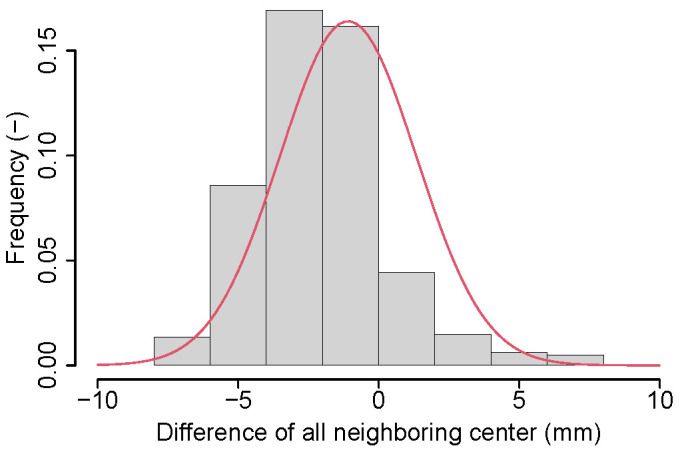
The histogram of the difference in the distance between each pair of neighboring vertebral centers projected on the z-axis across the training data set. Both lumbar and thoracic vertebrae are included.

**Figure 12:**
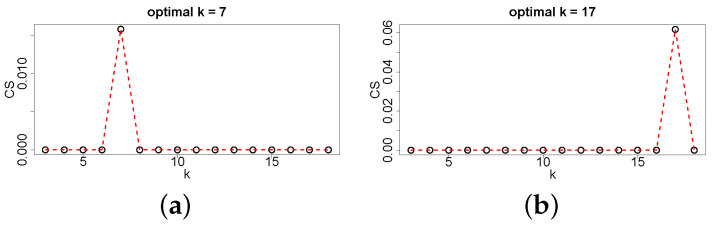
Two examples (**a**,**b**) of selecting the optimal value for *k* using the proposed objective function.

For objective function design, we observed that the distance between each pair of neighboring lumbar vertebra centers projected on the z-axis roughly follows a Gaussian distribution (Figure 8). However, this is not the case when considering lumbar and thoracic vertebrae due to changing vertebrae size and proximity differences (Figure 9). Consequently, we propose to utilize multiple Gaussian functions. First, a Gaussian is fit to the distance between each individual pair of vertebral centers by using a maximum likelihood estimation (MLE). Specifically, a dedicated Gaussian function is fit to each of the 17 distributions in the boxplot shown in Figure 10, resulting in a total of 17 Gaussian functions to represent the entire distance distribution. To make the objective function more robust, the difference in the distance between each pair of neighboring vertebral centers projected on the z-axis is also utilized. As shown in Figure 11, the difference in each neighboring center distance roughly follows a Gaussian distribution, which is in contrast to the neighboring vertebral center distance in Figure 9. Therefore, the objective function is focused on more local properties. Specifically, we focus on every three consecutive vertebral centers to formulate the objective function. For the three consecutive centers, there are two distances between the two pairs of centers and one difference between the two distances. When weighted by their corresponding Gaussian functions, a larger value indicates a better match to the expected distribution. Thus, the three Gaussian function values are multiplied, resulting in an objective function value for each *k*. While the products of all possible options of consecutive three centers form a set, we only utilize the consecutive part of the set which has the minimum product value because it represents the worst match of the set. Finally, $k_{opti}$ is chosen to have the maximum clustering score (CS):

(1) $$k_{opti}=\underset{k\in [3,19]}{\arg\max}\left\{CS\left(k\right)\right\}$$

where $CS\left(k\right)=\underset{i=1\cdots k-2}{min}\left\{G_{i}^{dst}\left(D_{i}\right)G_{i+1}^{dst}\left(D_{i+1}\right)G^{diff}\left(D_{i+1}-D_{i}\right)\right\}$ represents the objective function, $D_{i}$ is the distance between the *i*-th pair of predicted neighboring centers projected on the z-axis, $G_{i}^{dst}\left(\right)$ is the Gaussian weight for $D_{i}$, and $G^{diff}\left(\right)$ is the Gaussian weight for the difference in neighboring distances $D_{i+1}-D_{i}$. Two examples of objective function evaluation are given in Figure 12, and one can observe that the objective function is quite selective.

### 2.5. Identifying Individual Vertebrae and Segmentation Post-Processing

To separate connected vertebrae, the following processing steps are performed. Once the $k_{opti}$ vertebra centers are generated, a signed distance transform is computed only on the body label map to generate a distance map where its magnitude represents the distance from the segmentation boundaries and its sign indicates whether a voxel is inside or outside the segmentation, whereby the inside values are set to be negative. Then, each center is shifted to its local minimum on the distance map with a kernel size of 5×5×5 to refine the center locations (Figure 6d). Finally, a seeded watershed algorithm using the $k_{opti}$ center points as markers is applied on the distance map to split the segmentation into regions with different labels. A morphological dilation is performed after the seeded watershed for each labeled vertebra to recover the boundaries. Moreover, to prevent over-dilation, all labeled voxels that are not in the original body-boundary label map are excluded. These segmented regions are then relabeled from bottom to top (Figure 6e).

When looking at the output of the localization and segmentation method, we sometimes observe that some upper thoracic vertebrae are missing. This is either because (a) the smaller sizes of upper thoracic vertebrae produce a smaller number of localization votes, compared to lumbar or lower thoracic vertebrae with larger sizes, which results in less-accurate cluster centers, or (b) vertebrae are deformed/collapsed, resulting in the predicted center being located outside the vertebral body. To solve these two problems, we introduce a post-processing algorithm for recovering missed vertebrae. The algorithm consists of the following steps.

1. To identify only unlabeled vertebrae, the current labeled segmentation (with *k* labeled vertebrae) is applied as a mask to the three-label segmentation CNN output.
2. A connected component labeling algorithm is applied to the unlabeled parts, and the components are sorted.
3. Each connected component from step (2) is processed from largest to smallest as follows. First, the center coordinate of the component (C) is found. Second, the coordinate C together with the centers of all currently segmented vertebrae are assessed by utilizing Equation (1). If CS(k+1)>=CS(k), the component is added to the current segmentation, and k is updated as k=k+1.
4. Step (3) is repeated until either of the two following conditions are met: (a) the total number of labeled vertebrae (k) is larger than 18, which is the maximum number of vertebrae that are considered, or (b) all connected components from step (2) are processed.
5. All vertebrae in the current segmentation are relabeled from bottom to top to form the final labeled segmentation.

**Figure 13:**
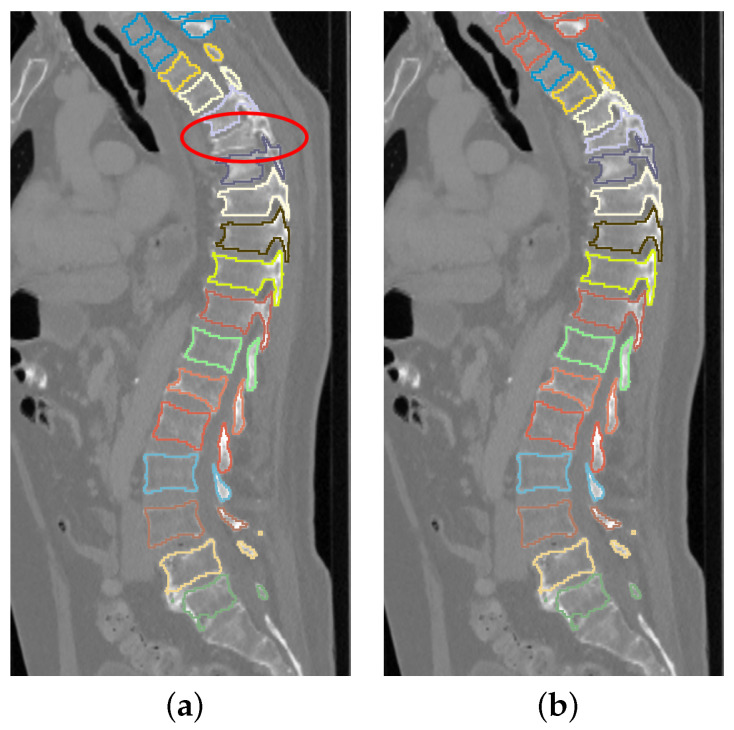
An example showing the segmentation results before (**a**) and after (**b**) the post-processing step, where the missing collapsed vertebra (marked by red oval) is recovered.

An example case with a collapsed vertebra that shows the effect of the post-processing algorithm can be found in Figure 13. As can be seen, the missing collapsed vertebra is recovered. In addition, for each individual vertebra label, another post-processing step is applied to remove potential islands and holes. First, a connected component labeling algorithm is applied to the segmentation output, and all components except the largest are removed. Then, the resulting label map is inverted, and the two previous steps are repeated on the inverted label map. Finally, the resulting label map is inverted again to produce the final segmentation label map.

## 3. Experimental Setup

### 3.1. Image Data

Two diverse types of image data are used in our experiments. The Iowa image data are representative of our target application—BMS IMRT. To assess the robustness and enable a comparison with other methods published, we also utilize VerSe2020 image data. Both image data sets are described in detail below.

#### 3.1.1. Iowa Data

The Iowa image data represent the subset of CT scans used for our pelvis segmentation experiments described in [31], for which a reference vertebrae segmentation is available. The segmentations were produced following current clinical practice. First, global thresholding was applied. Second, manual editing was performed to correct major errors. Overall, 26 planning CT scans with reference segmentations of lumbar vertebrae (L1–L5) are utilized. Combined, the CT scans include 129 vertebrae. The 26 planning CT scans had voxel sizes ranging from 0.977×0.977×2 mm to 1.367×1.367×2 mm, and the volume (matrix) size ranged from 512×512×174 to 512×512×353 voxels. All CT scans with a different voxel size than the median (0.977×0.977×2) were resampled to match the median size to avoid unnecessary blurring due to interpolation. Then, the scans were cropped from the axial center to maintain the 512×512 axial matrix size, followed by clipping to the [0.5, 99.5] percentiles of the HU value range of the entire data set to avoid extreme values. Finally, a Z-score normalization using the mean and standard deviation of the entire data set is implemented.

#### 3.1.2. Verse2020 Data

The VerSe2020 subject-based data structure [34] is a publicly available vertebra data collection that was used in conjunction with the VerSe’20 segmentation challenge [18,35,36]. Specifically, the VerSe2020 data set [34] contains 214 CT scans that were utilized to expand the VerSe’19 challenge data collection. It contains CT scans produced with several different scanners and includes varying image qualities as well as a wide range of data and voxel sizes. Thus, it is more diverse compared to Iowa image data (Section 3.1.1). Vertebra segmentation was performed by using a human–hybrid approach [18]. Note that we resampled all scans to the same voxel size of 0.977×0.977×2 mm to match the size of the voxels of the Iowa scans. Furthermore, each scan of VerSe2020 was clipped to the [0.05, 99.5] percentiles of the HU value range of the dedicated training data set, followed by a Z-score normalization using the mean and standard deviation of the training data set as well.

The data curators of the VerSe2020 data split them into training (n = 61), validation (n = 80), and test (n = 73) sets. In conjunction with the VerSe’20 segmentation challenge, the validation set was used as public and the test set as hidden test data. We follow this schema and utilize the hidden test data the same way as the validation data set, namely as an additional set of independent data for performance assessments. Furthermore, the VerSe2020 data set contains CT scans of the entire spine or portions thereof. However, our focus is on oncology applications in the pelvic region with a focus on lumbar and (lower) thoracic vertebrae. Consequently, some scans were excluded because most of the vertebrae shown are cervical vertebrae (validation set: 10 scans and test set: 14 scans). Thus, the available number of scans for the validation and test set were 70 and 59, respectively. The number of vertebrae in these sets was 873 and 761, respectively. Thus, these two sets include 1634 vertebrae.

### 3.2. Reference Segmentation

Both data sets utilized include a segmentation of vertebrae (i.e., one mask label per vertebra). To generate the three segmentation structures (the inner body of vertebra, vertebra boundary, and background) required for training, a morphological erosion operation using a spherical kernel with a radius of one was applied to the original label maps. The remainder of the erosion was used to generate the inner body label, while the eroded portion was assigned the boundary label.

### 3.3. Performance Metrics

The main motivation behind our approach is to facilitate the radiation oncology treatment of cancers in the pelvic region. For this application, an accurate segmentation of individual lumbar and thoracic vertebrae is desirable. Thus, to assess the segmentation performance, the Dice coefficient (Dice) [37] as well as signed ($d_{s}$) and unsigned ($d_{u}$) distance errors [38] were utilized on a per vertebrae basis. Note that for calculating $d_{s}$, segmentation surface points inside the reference were assigned a negative distance. In addition, the Hausdorff distance (HD) [38] is utilized.

Because the anatomical labeling of individual vertebrae (e.g., L5,…, L1, T12,…) is not a major objective of the presented work, a simple numbering scheme, starting with the vertebrae next to the pelvis (VerSe2020 alternative if the pelvis is not imaged: the lowest vertebra imaged), is sufficient to differentiate between vertebrae. Because the VerSe2020 CT scans can completely, partially, or not at all depict the pelvis, a correspondence between the segmented and reference segmentation vertebrae needs to be established before assessing the segmentation performance. For this purpose, the overlap between the segmentation result and reference is calculated on a vertebrae basis. Subsequently, an assignment is made based on maximum overlap. To account for missing vertebrae (a false negative, FN) or erroneously segmented other structures (a false positive, FP), the number of false negatives and positives is reported for the VerSe2020 data, in combination with the number of true positive (TP) cases. In addition, the resulting F1-scores ($\frac{2TP}{(2TP+FP+FN}$) are reported.

## 4. Results

### 4.1. Iowa Data

For the Iowa data set, five segmentation–localization models were trained in each of the three independent experiments, and the best-performing model out of each five-fold cross-validation setups (Section 2.2) is utilized. Table 2 summarizes the resulting performance metrics. We note that $d_{s}$ is significantly larger than 0 (p<0.05), meaning there is a positive bias and segmentation results tend to be larger than the ground truth.

**Figure 14:**
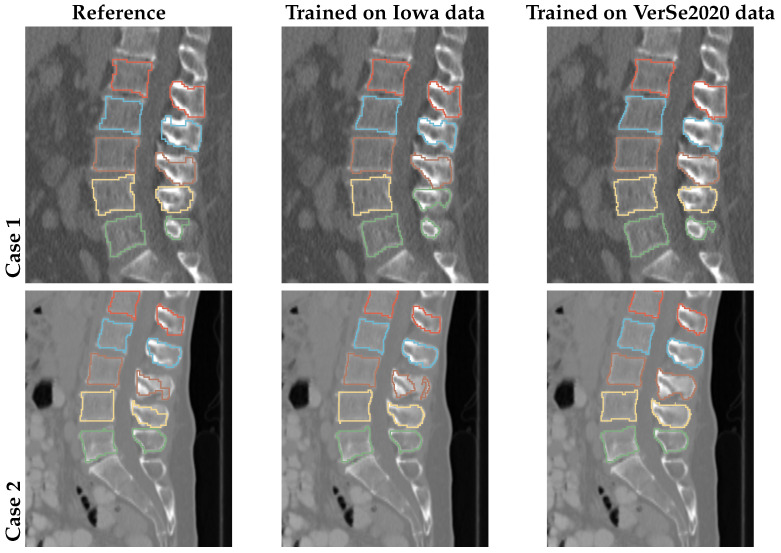
Examples of sagittal slices of the Iowa data set ground truth and corresponding segmentation output from models trained on Iowa and VerSe2020 data, respectively.

Typical examples of our method trained on and applied to Iowa CT images are depicted in the middle column of Figure 14. For comparison, the reference segmentation is provided in the first column of Figure 14. As can be seen, the reference segmentation, which was produced by using the currently clinically utilized approach of thresholding and manual editing, can be locally inaccurate. In contrast, the network produces a more visually appealing result. For comparison, we trained our approach on VerSe2020 training data and applied it to the same scans (the last column in Figure 14), resulting in better segmentations with less local errors. Thus, we conclude that the reference segmentations provided for VerSe2020 are of higher quality than for the Iowa data set. The results in Figure 14 demonstrate that (a) the network generalizes well even if a limited training set is available and (b) it scales in segmentation performance with reference data size and quality.

### 4.2. VerSe2020 Data

As mentioned in Section 2.2, both VerSe2020 validation and test sets are regarded as independent sets for performance analysis. Similar to the analysis of Iowa data, we selected the best-performing model out of the five-fold cross-validation setup. Furthermore, the results are reported for both categories: (A) cases where the pelvis segmentation succeeded and (B) cases where the pelvis segmentation failed, or the pelvis is only partially or not imaged. Table 3 provides a summary of all the assessed segmentation error metrics. Note that the value of $d_{s}$ for both the “validation” and “test” sets are significantly larger than 0 (p<0.05), meaning there is a positive bias and segmentation results tend to be larger than the ground truth. Table 4 provides TP, FP, and FN numbers and the resulting F1-scores of the segmentation results.

**Figure 15:**
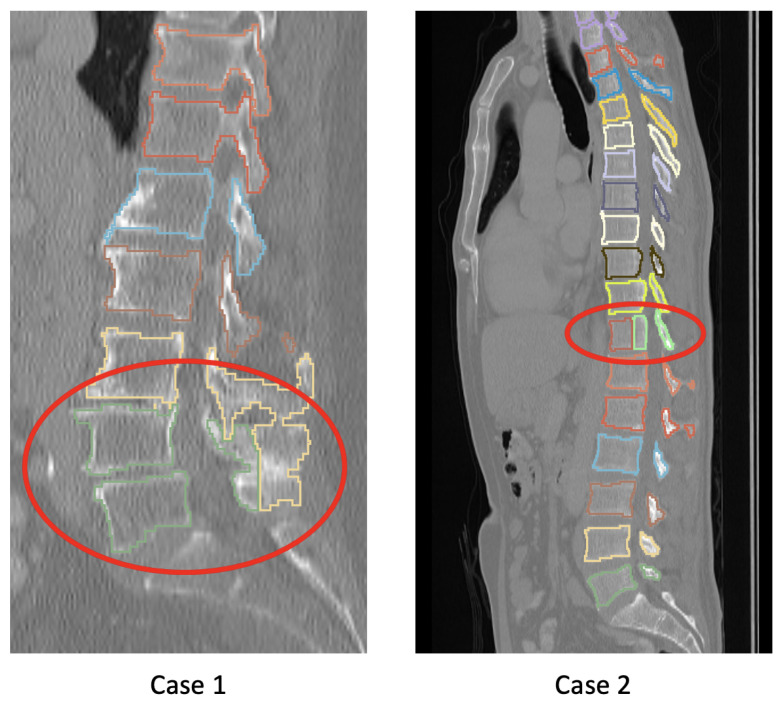
Examples of infrequent segmentation issues caused by low bone density. See text in Section 4.2 for details.

When combining validation and test sets as well as both categories (i.e., 129 CT scans showing 1634 vertebrae), the following results were obtained: Dice = 0.940±0.065, $d_{u}$ = 0.159±0.407 mm, $d_{s}$ = 0.109±0.301 mm, and HD = 5.482±6.001 mm. If the bone density is quite low, neighboring vertebrae can be fused (Case 1 in Figure 15) or a vertebra can get split into two or more parts (Case 2 in Figure 15). However, these cases are quite infrequent.

Our method utilizes three labels (boundary, inner body, and background) to segment and separate individual vertebrae. In this context, the Dice coefficient for the inner vertebra body was 0.96, and for the vertebra boundary, 0.80. Given a pelvis segmentation (average computing time: 250 s), our approach takes between 35 and 110 s to locate and segment vertebrae, depending on the data set size.

**Figure 16:**
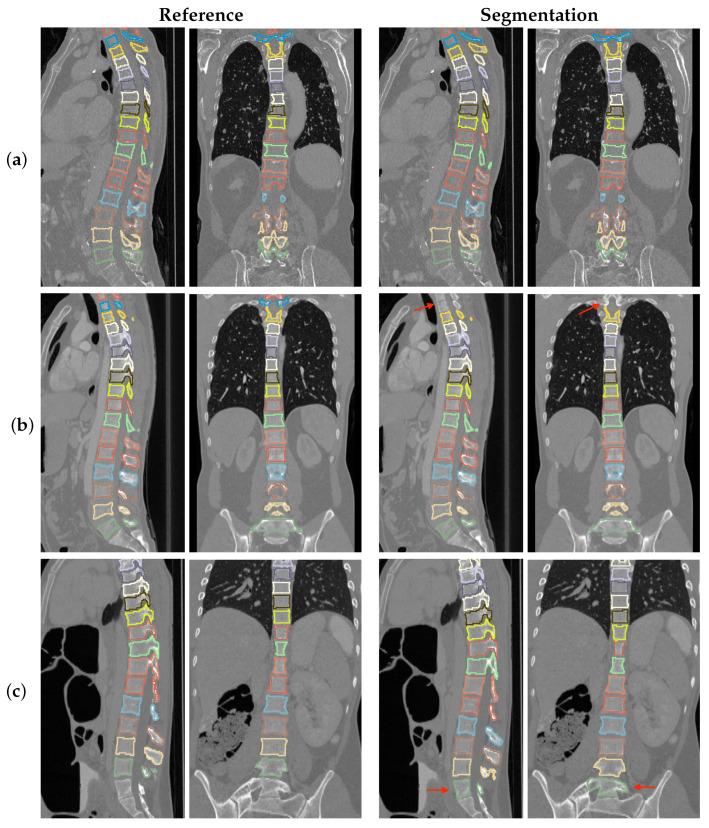
Comparison of ground truth vs. automated segmentation results of category (A) VerSe2020 data. (**a**) All vertebrae are successfully segmented. (**b**) T1 and T2 (red arrow) are missing in the automated segmentation result. (**c**) S1 (red arrow) is included in the automated segmentation result.

**Figure 17:**
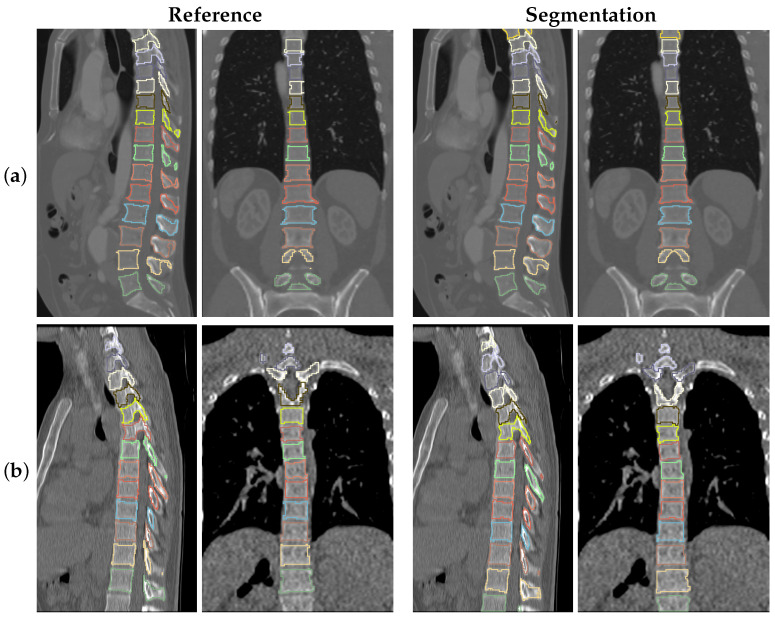
Comparison of ground truth vs. automated segmentation results of category (B) VerSe2020 data. (**a**) The pelvis is partially imaged. (**b**) The pelvis is not imaged.

Overall, the number of FPs was 28 (1.714%) and the number of FNs was 13 (0.796%). In this context, we note that FPs are simpler to correct (i.e., delete) than FNs. Examples showing a visual comparison between ground truth and automated segmentation results for category (A) are shown in Figure 16. Examples of category (B) results are provided in Figure 17. In this context, it is known that the VerSe’20 challenge data have an over-representation of “rare anatomical anomalies in the form of transitional vertebrae” like T13 and L6 [18]. Compared to VerSe’19, the predecessor of VerSe’20, this was achieved by adding VerSe2020 data. In our experiments, we observed only one FN related to a missing L6 segmentation. Furthermore, most of the FN cases stem from missing T1 and T2 (Figure 16b). The vast majority of FP cases can be explained as follows. Compared to the Iowa data, VerSe2020 is more diverse in terms of patients’ age, including more younger patients. For these patients, sacral S1 can appear with strong, well-separated boundaries. Our review of FP cases showed that most FP cases represent S1, which are not part of the VerSe2020 reference segmentation. An example of such a case is given in Figure 16c. Thus, we speculate that a more diverse training set for the pelvis CNN will address this issue.

## 5. Discussion

### 5.1. Segmentation Performance

Our method was trained and tested on two different data sets, achieving average Dice coefficients of 0.921 on the small-sized Iowa and 0.940 on the large-sized VerSe2020 data set. Similar trends were observable for the distance-based error measures. However, we note that even on the small-sized Iowa data set, the signed and unsigned distance errors are already well below the size of a voxel. Furthermore, the average Hausdorff distance of 8.731 mm can be explained as follows. The ground truth segmentations provided for the Iowa data set were produced following the same procedure as used in physician clinical practice and did show local segmentation inaccuracies. Despite this issue, our approach has demonstrated its ability to generalize and produce better-matching vertebrae contours compared to the reference (Figure 14). Thus, one advantage of our approach is that it showed capacity to work well when trained on a small, imperfect data set (Iowa) and a large data set (VerSe2020). Specifically, the performance on the Iowa data was quite good and scaled with the availability of more training data of VerSe2020 accordingly.

On VerSe2020 data, the performance achieved in category (A) (cases where the pelvis segmentation succeeded) and category (B) (cases where the pelvis segmentation has failed) data is quite similar. This is expected because of the selective response of the developed network (Figure 2) to vertebra structures. In this context, we note that the use of pelvis segmentation to generate a VOI for subsequent processing is mainly motivated by achieving a reduction in computing time.

A comparison of our approach to other methods that were evaluated on VerSe data sets is provided in Table 5. The best-performing method of the VerSe’20 challenge had a Dice coefficient of 0.9172 and 0.9123 on the validation and test sets, respectively [18]. We note that our approach shows a better performance on both data sets utilized in our work. However, this is only a rough comparison, because we are only segmenting lumbar and thoracic vertebrae, not including cervical vertebrae. Similarly, when comparing our results to the results of methods that were published after the VerSe’20 challenge and used VerSe data, our approach performed more favorably. We attribute this to (a) using segmentation results from all volume patches processed and (b) utilizing a post-processing method.

Furthermore, we note that the recently introduced ViT-based single-network approach proposed by You et al. [30] had a quite low Dice coefficient when compared to our single-network method as well as other top-performing multi-network methods listed in Table 5. Again, this demonstrates the advantage of our approach, consisting of a balanced combination of deep learning and traditional computer vision methods for post-processing.

### 5.2. Current Limitations and Future Work

The focus of the presented work was on accurately segmenting lumbar and neighboring thoracic vertebrae. If needed, cervical vertebrae can be easily integrated into our vertebrae center localization approach, which models the characteristics of vertebrae individually. In addition, relevant training data for cervical vertebrae will be required.

A precise labeling of each individual vertebra was not crucial for our application in radiation oncology. Thus, the focus of our method was to accurately locate and segment each individual vertebra, and a simple labeling of each vertebra from bottom to top was utilized. If needed, a more refined vertebrae labeling can be applied after utilizing our segmentation algorithm. Such an algorithm will require global information of the entire CT scan to accurately assign vertebrae labels (e.g., T12, T13, or L1). Furthermore, in our application, a pelvis segmentation is required, which was used to define a VOI for locating individual vertebrae. For this purpose, we utilized our single-object combined localization and segmentation approach in combination with a random sampling strategy [31]. If needed, similar ideas as discussed in Xiong et al. [31] can be adapted to localize and segment vertebrae without a preceding pelvis segmentation. In future work, we plan to generalize our approach to handle multiple different objects like vertebrae, ribs, clavicle, and other bone structures.

## 6. Conclusions

We presented a novel approach for the segmentation of individual lumbar and thoracic vertebra in CT scans. Our method utilizes a multi-object combined localization and segmentation approach. The segmentation results showed superior performance in individually segmenting lumbar and thoracic vertebrae. This was achieved in a diverse imaging data set with a variety of scanners and acquisition parameters. The proposed approach has several clinically relevant applications, including facilitating bone-marrow-preserving adaptive radiation therapy and bone marrow dose calculation for radiopharmaceutical therapy. Given the performance of the three-label (boundary, inner body, and background) approach to segment vertebra, our method has the potential to facilitate applications requiring segmenting vertebral spongiosa from the cortical bone, which could be even more useful for radiopharmaceutical dosimetry.

## Figures and Tables

**Table 1:** Summary of pelvis segmentation on VerSe2020 image data.

	Validation	Test
Full pelvis, segmentation succeeded	57	45
Full pelvis, segmentation failed	3	2
Partial or no pelvis	10	12
Total	70	59

**Table 2:** Summary of segmentation performance metrics on the Iowa data set.

Error Metric		Mean ± Std	Median
Dice	(-)	0.921 ± 0.047	0.936
$d_{s}$	(mm)	0.271 ± 0.748	0.067
$d_{u}$	(mm)	0.564 ± 0.757	0.337
HD	(mm)	8.731 ± 7.410	6.035

**Table 3:** Summary of segmentation results on VerSe2020 data for both categories.

Category (A)					
		Validation Set		Test Set	
Error Metric		Mean ± Std	Median	Mean ± Std	Median
Dice	(-)	0.936 ± 0.082	0.953	0.946 ± 0.039	0.955
$d_{s}$	(mm)	0.118 ± 0.375	0.047	0.070 ± 0.168	0.045
$d_{u}$	(mm)	0.134 ± 0.377	0.055	0.086 ± 0.143	0.055
HD	(mm)	6.171 ± 7.067	3.906	4.851 ± 4.876	3.410
**Category (B)**					
		**Validation Set**		**Test Set**	
**Error Metric**		**Mean ± Std**	**Median**	**Mean ± Std**	**Median**
Dice	(-)	0.928 ± 0.070	0.946	0.945 ± 0.046	0.954
$d_{s}$	(mm)	0.203 ± 0.411	0.109	0.110 ± 0.095	0.089
$d_{u}$	(mm)	0.398 ± 0.745	0.188	0.263 ± 0.489	0.163
HD	(mm)	6.108 ± 6.200	4.000	4.212 ± 3.782	3.088

**Table 4:** Summary of true positive (TP), false positive (FP), and false negative (FN) numbers as well as F1-scores achieved on VerSe2020 data for both categories.

Category (A)		
	Validation Set (n = 696)	Test Set (n = 588)
Number of TP	684	577
Number of FP	7	10
Number of FN	5	1
F1-score	0.991	0.991
**Category (B)**		
	**Validation Set (n = 177)**	**Test Set (n = 173)**
Number of TP	167	165
Number of FP	7	4
Number of FN	3	4
F1-score	0.971	0.976

**Table 5:** Performance comparison of vertebrae segmentation methods that have used VerSe image data. † Notes that You et al. report HD_95%_ instead of the standard HD to alleviate the effect of outliers [30].

	Validation Set (“Public”)		Test Set (“Hidden”)		
Approach	Dice (-)	HD (mm)	Dice (-)	HD (mm)	Notes/Comments
(a) Participants VerSe’19 challenge (see Sekuboyina et al. [18] for details)					
Payer C.	0.9090	6.35	0.8980	7.08	
Lessmann N.	0.8508	8.58	0.8576	8.20	
Chen M.	0.9301	6.39	0.8256	9.98	
Amiranashvili T.	0.6702	17.35	0.6896	17.81	
Dong Y.	0.7674	14.09	0.6751	26.46	
Angermann C.	0.4314	44.27	0.4640	41.64	
Kirszenberg A.	0.1371	77.48	0.3564	65.51	
Jiang T.	0.8270	11.22	-	-	
Wang X.	0.7188	24.59	-	-	
Brown K.	0.6269	35.90	-	-	
Hu Y.	0.8407	12.79	0.8182	29.94	
Sekuboyina A.	0.8306	12.11	0.8318	9.94	
(b) Participants VerSe’20 challenge (see Sekuboyina et al. [18] for details)					
Chen D.	0.9172	6.14	0.9123	7.15	
Payer C.	0.9165	5.80	0.8971	6.06	
Zhang A.	0.8882	7.62	0.8936	7.92	
Yeah T.	0.8888	9.57	0.8791	8.41	
Xiangshang Z.	0.8358	15.19	0.8507	12.99	
Hou F.	0.8399	8.10	0.8492	8.08	
Zeng C.	0.8399	9.58	0.8439	8.73	
Huang Z.	0.8075	34.06	0.8169	15.75	
Netherton T.	0.7516	13.56	0.7826	14.06	
Huynh L.	0.6248	20.29	0.6523	20.35	
Jakubicek R.	0.7317	17.26	0.5297	20.30	
Mulay S.	0.5818	99.75	-	-	
Paetzold J.	0.1060	166.55	0.2549	240.61	
Sekuboyina A.	0.7805	10.99	0.7952	11.61	
(c) Recently published papers that use VerSe data sets for performance evaluation					
Lu et al. [25]	Dice: 0.904		HD: -		Based on 156 CT scans of VerSe 2020 data set,
					did not specify public/hidden,
					used lumbar vertebrae only,
					mean of results reported for L1 to L5 is shown
Meng et al. [28]	0.9253	7.03	0.9111	6.69	Based on 200 VerSe’20 CT scans
Qadri et al. [27]	Dice: 90.2		HD: -		VerSe data subset mixed with other data sets
You et al. [30]	0.8639	3.41 †	0.8654	3.66 †	Based on VerSe’19 data set
You et al. [30]	0.8453	6.34 †	0.8686	4.60 †	Based on VerSe’20 data set
(d) Our approach					
Category (A)	0.936	6.171	0.946	4.851	See Section 3.1.2 for details
Category (B)	0.928	6.108	0.945	4.212	See Section 3.1.2 for details

## Data Availability

The VerSe2020 data set is publicly available [34]. Iowa data are not shared.

## References

[1] McGuire S.M., Bhatia S.K., Sun W., Jacobson G.M., Menda Y., Ponto L.L., Smith B.J., Gross B.A., Bayouth J.E., Sunderland J.J. (2016). Using [(18)F]Fluorothymidine Imaged With Positron Emission Tomography to Quantify and Reduce Hematologic Toxicity Due to Chemoradiation Therapy for Pelvic Cancer Patients. Int. J. Radiat. Oncol. Biol. Phys..

[2] Mastmeyer A., Engelke K., Fuchs C., Kalender W.A. (2006). A hierarchical 3D segmentation method and the definition of vertebral body coordinate systems for QCT of the lumbar spine. Med. Image Anal..

[3] Burnett S.S., Starkschalla G., Stevens C.W., Liao Z. (2004). A deformable-model approach to semi-automatic segmentation of CT images demonstrated by application to the spinal canal. Med. Phys..

[4] Shen H., Litvin A., Alvino C. Localized priors for the precise segmentation of individual vertebras from CT volume data. Proceedings of the International Conference on Medical Image Computing and Computer-Assisted Intervention.

[5] Kelm B.M., Zhou S.K., Suehling M., Zheng Y., Wels M., Comaniciu D., Menze B., Langs G., Tu Z., Criminisi A. (2011). Detection of 3D Spinal Geometry Using Iterated Marginal Space Learning. Medical Computer Vision. Recognition Techniques and Applications in Medical Imaging.

[6] Howe B., Gururajan A., Sari-Sarraf H., Long L. Hierarchical segmentation of cervical and lumbar vertebrae using a customized generalized Hough transform and extensions to active appearance models. Proceedings of the 6th IEEE Southwest Symposium on Image Analysis and Interpretation.

[7] Kim K., Lee S. (2017). Vertebrae localization in CT using both local and global symmetry features. Computerized Medical Imaging and Graphics.

[8] Chu C., Belavý D.L., Armbrecht G., Bansmann M., Felsenberg D., Zheng G. (2015). Fully Automatic Localization and Segmentation of 3D Vertebral Bodies from CT/MR Images via a Learning-Based Method. PLoS ONE.

[9] Bromiley P.A., Kariki E.P., Adams J.E., Cootes T.F. (2016). Fully Automatic Localisation of Vertebrae in CT Images Using Random Forest Regression Voting. Proceedings of the Computational Methods and Clinical Applications for Spine Imaging.

[10] Sekuboyina A., Rempfler M., Kukačka J., Tetteh G., Valentinitsch A., Kirschke J.S., Menze B.H. (2018). Btrfly Net: Vertebrae Labelling with Energy-Based Adversarial Learning of Local Spine Prior. Proceedings of the Medical Image Computing and Computer Assisted Intervention—MICCAI 2018.

[11] Mader A.O., Lorenz C., von Berg J., Meyer C., Shen D., Liu T., Peters T.M., Staib L.H., Essert C., Zhou S., Yap P.T., Khan A. (2019). Automatically Localizing a Large Set of Spatially Correlated Key Points: A Case Study in Spine Imaging. Proceedings of the Medical Image Computing and Computer Assisted Intervention—MICCAI 2019.

[12] McCouat J., Glocker B. (2019). Vertebrae Detection and Localization in CT with Two-Stage CNNs and Dense Annotations. arXiv.

[13] Kang Y., Engelke K., Kalender W.A. (2003). A new accurate and precise 3-D segmentation method for skeletal structures in volumetric CT data. IEEE Trans. Med. Imaging.

[14] Aslan M.S., Ali A., Rara H., Farag A.A. An automated vertebra identification and segmentation in CT images. Proceedings of the 2010 IEEE International Conference on Image Processing.

[15] Hammernik K., Ebner T., Štern D., Urschler M., Pock T. (2015). Vertebrae segmentation in 3D CT images based on a variational framework. Recent Advances in Computational Methods and Clinical Applications for Spine Imaging.

[16] Korez R., Ibragimov B., Likar B., Pernuš F., Vrtovec T. (2015). Interpolation-Based Shape-Constrained Deformable Model Approach
for Segmentation of Vertebrae from CT Spine Images. Recent Advances in Computational Methods and Clinical Applications for Spine Imaging.

[17] Ibragimov B., Likar B., Pernuš F., Vrtovec T. (2014). Shape representation for efficient landmark-based segmentation in 3-D. IEEE Trans. Med. Imaging.

[18] Sekuboyina A., Husseini M.E., Bayat A., Löffler M., Liebl H., Li H., Tetteh G., Kukačka J., Payer G., Štern D. (2021). VerSe: A Vertebrae labelling and segmentation benchmark for multi-detector CT images. Med. Image Anal..

[19] Yao J., Burns J.E., Forsberg D., Seitel A., Rasoulian A., Abolmaesumi P., Hammernik K., Urschler M., Ibragimov B., Korez R. (2016). A multi-center milestone study of clinical vertebral CT segmentation. Comput. Med. Imaging Graph..

[20] Yao J., Burns J.E., Munoz H., Summers R.M., Ayache N., Delingette H., Golland P., Mori K. (2012). Detection of Vertebral Body Fractures Based on Cortical Shell Unwrapping. Proceedings of the Medical Image Computing and Computer-Assisted Intervention—MICCAI 2012.

[21] Payer C., Stern D., Bischof H., Urschler M., Farinella G., Radeva P., Braz J. (2020). Coarse to Fine Vertebrae Localization and Segmentation with Spatial Configuration-Net and U-Net. Proceedings of the 16th International Joint Conference on Computer Vision, Imaging and Computer Graphics Theory and Applications: VISIGRAPP 2021.

[22] Lessmann N., van Ginneken B., de Jong P.A., Išgum I. (2019). Iterative fully convolutional neural networks for automatic vertebra segmentation and identification. Med. Image Anal..

[23] Chen D., Bai Y., Zhao W., Ament S., Gregoire J., Gomes C. Deep Reasoning Networks for Unsupervised Pattern De-mixing with Constraint Reasoning. Proceedings of the 37th International Conference on Machine Learning.

[24] Cheng P., Yang Y., Yu H., He Y. (2021). Automatic vertebrae localization and segmentation in CT with a two-stage Dense-U-Net. Sci. Rep..

[25] Lu H., Li M., Yu K., Zhang Y., Yu L. (2023). Lumbar spine segmentation method based on deep learning. J. Appl. Clin. Med. Phys..

[26] Wu Z., Xia G., Zhang X., Zhou F., Ling J., Ni X., Li Y. (2022). A novel 3D lumbar vertebrae location and segmentation method based on the fusion envelope of 2D hybrid visual projection images. Comput. Biol. Med..

[27] Qadri S.F., Lin H., Shen L., Ahmad M., Qadri S., Khan S., Khan M., Zareen S.S., Akbar M.A., Bin Heyat M.B. (2023). CT-Based Automatic Spine Segmentation Using Patch-Based Deep Learning. Int. J. Intell. Syst..

[28] Meng D., Boyer E., Pujades S. (2023). Vertebrae localization, segmentation and identification using a graph optimization and an anatomic consistency cycle. Comput. Med. Imaging Graph..

[29] Dosovitskiy A., Beyer L., Kolesnikov A., Weissenborn D., Zhai X., Unterthiner T., Dehghani M., Minderer M., Heigold G., Gelly S. (2020). An Image is Worth 16 × 16 Words: Transformers for Image Recognition at Scale. arXiv.

[30] You X., Gu Y., Liu Y., Lu S., Tang X., Yang J. (2023). VerteFormer: A single-staged Transformer network for vertebrae segmentation from CT images with arbitrary field of views. Med. Phys..

[31] Xiong X., Smith B.J., Graves S.A., Sunderland J.J., Graham M.M., Gross B.A., Buatti J.M., Beichel R.R. (2022). Quantification of uptake in pelvis F-18 FLT PET-CT images using a 3D localization and segmentation CNN. Med. Phys..

[32] Çiçek Ö., Abdulkadir A., Lienkamp S.S., Brox T., Ronneberger O., Ourselin S., Joskowicz L., Sabuncu M.R., Unal G., Wells W. (2016). 3D U-Net: Learning Dense Volumetric Segmentation from Sparse Annotation. Proceedings of the Medical Image Computing and Computer-Assisted Intervention—MICCAI 2016.

[33] Ulyanov D., Vedaldi A., Lempitsky V.S. (2016). Instance Normalization: The Missing Ingredient for Fast Stylization. arXiv.

[34] Kirschke J.S., Löffler M., Sekuboyina A., Liebl H. VerSe2020 (Subject Based Data Structure). https://osf.io/4skx2/.

[35] Löffler M.T., Sekuboyina A., Jacob A., Grau A.L., Scharr A., El Husseini M., Kallweit M., Zimmer C., Baum T., Kirschke J.S. (2020). A Vertebral Segmentation Dataset with Fracture Grading. Radiol. Artif. Intell..

[36] Liebl H., Schinz D., Sekuboyina A., Malagutti L., Löffler M.T., Bayat A., El Husseini M., Tetteh G., Grau K., Niederreiter E. (2021). A computed tomography vertebral segmentation dataset with anatomical variations and multi-vendor scanner data. Sci. Data.

[37] Dice L.R. (1945). Measures of the Amount of Ecologic Association Between Species. Ecology.

[38] Sonka M., Hlavac V., Boyle R. (2007). Image Processing: Analysis and Machine Vision.

